# Current progress on identifying chemical constituents, bioactivities, and food-industry applications of pitaya (*Selenicereus* spp.) whole plant

**DOI:** 10.3389/fnut.2026.1886438

**Published:** 2026-07-03

**Authors:** Meixin Chen, Ruien Li, Lei Zhang, Baoyue Zhang, Xingyang Dong, Siyu Wang, Wei Zhang, Xueyuan Bai

**Affiliations:** 1 Northeast Asia Research Institute of Traditional Chinese Medicine, Changchun University of Chinese Medicine, Changchun, China; 2 Department of Endocrinology and Metabolism, The Affiliated Hospital of Changchun University of Chinese Medicine, Changchun, China; 3 Human Resources Department, The Affiliated Hospital of Changchun University of Chinese Medicine, Changchun, Jilin, China

**Keywords:** pitaya, *Selenicereus* spp., chemical composition, biological activity, mechanism of action, food application

## Abstract

Pitaya (*Selenicereus* spp.) is primarily consumed for its flesh, whereas by-products—including the peel, seeds, stem, and flower—are largely discarded, resulting in resource wastage and environmental burden. This review discusses its chemical composition, mechanism of action, biological activity, and applications in the food industry. By-products of Pitaya are rich in bioactive constituents, such as polyphenols, flavonoids, betalains, polysaccharides, and unsaturated fatty acids. They also exhibit biological activities including antioxidant, anti-inflammatory, hypolipidemic, and hypoglycemic effects. High-value utilization of these by-products is currently at a critical stage of industrial translation. Accordingly, this review systematically synthesizes research findings from multiple disciplines, including phytochemistry, food science, pharmacology, and materials science. These findings are integrated along three principal axes: chemical composition, biological activity, and food application, thereby bridging the logical gap between fundamental research and industrial application. Based on this, we identified the principal limitations of the current research: mechanistic analyses remain largely correlational, key pathway alterations are inconsistent across different experimental models, and the translational relationship between *in vitro* activity and *in vivo* efficacy remains unclear. Future studies should conduct standardized human clinical trials, employ multi-omics approaches to elucidate the interactions between bioactive constituents and the gut microbiota, optimize stabilization technologies such as encapsulation, and establish low-energy extraction processes and quality grading standards. This will facilitate the transformation of pitaya from a fresh fruit into functional food ingredients and bioactive materials.

## 1 Introduction

Pitaya (*Selenicereus* spp.), also known as red dragon fruit or dragon pearl fruit, is taxonomically classified under the genus *Hylocereus* of the family Cactaceae. According to the current taxonomic revision, it has been reclassified to the genus *Selenicereus* within the same family (1). It is a perennial climbing succulent shrub (2). Native to the tropical regions of Central America, it has become an important commodity in the global tropical fruit trade owing to its distinctive appearance, sweet and refreshing flavor, and abundant nutritional value (3). Through long-term evolutionary adaptation to tropical climatic conditions, pitaya is now cultivated at scale in countries, including China, Egypt, the Philippines, and Germany (4). Currently, commercially cultivated varieties are primarily classified into three types: red-skinned, white-fleshed pitaya (*Selenicereus undatus*); red-skinned, red-fleshed pitaya (*Selenicereus monacanthus*); and yellow-skinned, white-fleshed pitaya (*Selenicereus megalanthus*) (5). The pitaya industry has expanded rapidly owing to the growing consumer demand for health-promoting foods. Pitaya has entered global markets not only as a fresh fruit but also as a processed product, such as juice, dried fruit, and jam, which have become increasingly prevalent (6).

However, the pitaya industry faces the challenge of inefficient resource utilization alongside its rapid development. Currently, only the flesh is consumed, whereas by-products, including the peel, seeds, flower, and stem, are commonly discarded, resulting in resource wastage and environmental burden. Similar challenges exist for other tropical crops. For instance, *Opuntia ficus-indica* also faces by-product utilization issues. Studies have demonstrated that its peels and seeds are rich in phenolic compounds and unsaturated fatty acids, and high-value utilization can be achieved through green extraction technologies (7). This study provides important insights for the development of pitaya by-products.

Recent studies have revealed that pitaya by-products are abundant in diverse bioactive components with significant development potential. The peel generally exhibits higher phenolic and flavonoid content than the flesh and demonstrates stronger DPPH and ABTS radical-scavenging activities (8). Similar phenomena have been reported for other fruits and vegetables. For example, purple onion cultivars contain significantly higher total phenolic and anthocyanin contents than white cultivars, and these contents are positively correlated with antioxidant activity (9). This suggests that similar variations in active components may exist among different pitaya cultivars. Seed extracts also contain abundant flavonoids, phenolic acids, lignans, and coumarins (10). Regarding the extraction of active components from plant seeds, green technologies such as supercritical CO₂ extraction have demonstrated advantages in terms of efficiency and environmental sustainability (11), providing technical references for the extraction of pitaya seed oil. Betacyanins in the flesh not only confer the fruit its vivid coloration but also possess potent antioxidant activity and α-glucosidase inhibitory capacity (12). These functional components can exert synergistic antioxidant, anti-inflammatory, hypolipidemic, and hypoglycemic activities by modulating intestinal microbiota metabolism and producing SCFAs and vitamins. These mechanisms lay the foundation for the high-value application of pitaya bioactive components in functional foods, natural additives, and nutritional supplements.

Research on pitaya flesh has reached relative maturity in recent years. However, the high-value utilization of non-edible parts—including the peel, seeds, stem, and flower—remains at a critical stage of translation from fundamental research to industrial application (13, 14). A systematic review of the chemical compositional differences, structure–activity relationships, and bioactive mechanisms across different tissues of whole-plant pitaya, together with an exploration of its application potential in functional foods, natural food additives, and active packaging materials, holds important theoretical significance and practical value for promoting the transformation of the pitaya industry from fresh fruit sales toward comprehensive resource utilization, thereby enhancing industrial added value and sustainable development capacity (15, 16).

In this context, this review comprehensively examines the chemical constituents and bioactivities of whole-plant pitaya and its applications in the food industry. This study aimed to provide a scientific foundation for the in-depth development and integrated utilization of pitaya.

## 2 Chemical composition

**Figure 1:**
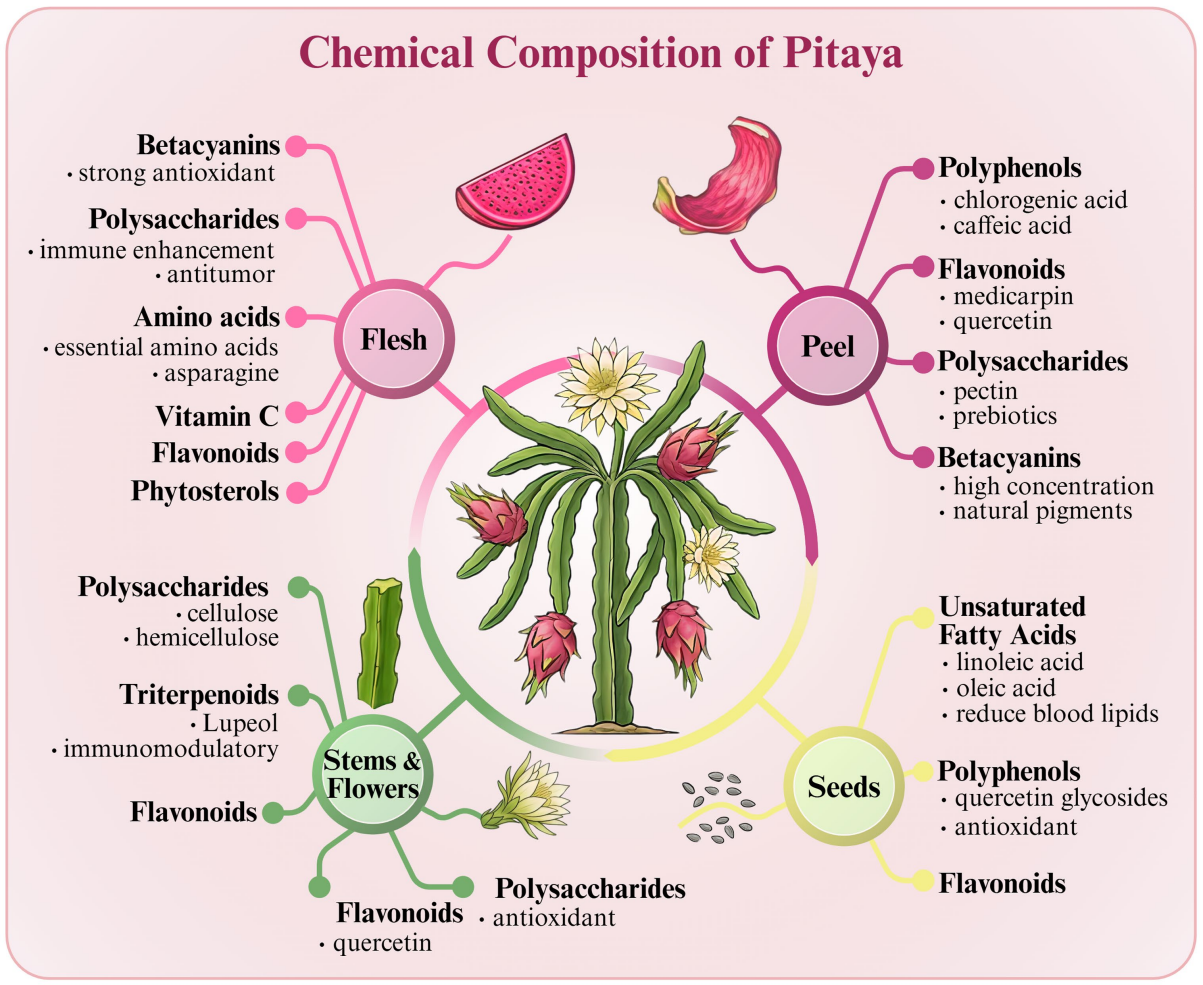
Chemical composition of pitaya (*Selenicereus* spp.) whole plant. Peel: polyphenols, flavonoids, pectic polysaccharides, betacyanins. Flesh: betacyanins, polysaccharides, amino acids, vitamin C, flavonoids, phytosterols. Seeds: unsaturated fatty acids, polyphenols, flavonoids. Stems and flowers: polysaccharides, triterpenoids, flavonoids (created in https://BioRender.com).

Pitaya is rich in diverse bioactive constituents, including betalains, phenolic compounds, flavonoids, polysaccharides, and organic acids (Figure 1). Significant differences in chemical composition exist among different varieties; for instance, white-fleshed and red-fleshed pitaya exhibit distinct profiles, with the red-fleshed variety demonstrating superior antioxidant properties attributable to its higher betalain content (17, 18). Various parts of the fruit—including the peel, flesh, and seeds—have been confirmed to contain high-value phytochemicals. Among these, the peel and seeds, as by-products, possess particularly prominent potential for resource utilization (10) (Table 1).

**Table 1:** Main bioactive compounds of different parts of pitaya (*Selenicereus* spp.).

Compound name	Plant part	Content	Reference
Betacyanins (including betanin, isobetanin, phyllocactin, isophyllocactin, 4′-O-malonylbetanin, etc.)	Peel	Betanin 9.44 mg/g in peel; total betacyanins 35.12 mg/g in peel	(31–33)
	Flesh	Total betacyanins 30.15 mg/g in pulp; betacyanins 15.18–20.03 mg/100 mL in pulp extract; betaxanthins 6.39–8.31 mg/100 mL in pulp extract; betacyanins 9.65 mg/100 g in dried peel powder	(40)
Anthocyanins (including cyanidin-3-glucoside, delphinidin-3-glucoside, cyanidin-3-O-rutinoside, etc.)	Peel	Total anthocyanins 19.21 mg/g in peel	(21, 35)
	Flesh	15.16 mg/g in pulp; detected in red-fleshed varieties, absent in white-fleshed varieties	(33)
Polyphenols (chlorogenic acid, caffeic acid, ferulic acid, p-coumaric acid, rutin, isoquercitrin, etc.)	Peel	Free polyphenols: chlorogenic acid, caffeic acid, ferulic acid, p-coumaric acid predominant in red-fleshed peel; chlorogenic acid, caffeic acid, rutin, isoquercitrin predominant in white-fleshed peel Bound polyphenols 2.38–2.45 mg GAE/g DW in peel	(19, 20)
	Flesh	Chlorogenic acid, caffeic acid, rutin, luteolin-7-glucoside, quercetin in flesh	(10, 36)
	Seeds	Total phenolic content 52–144 mg/g (mean 100.83 mg/g) in seeds	(48)
Flavonoids (quercetin-3-rhamnoside, myricetin, rutin, vitexin, catechin, epigallocatechin, luteolin, apigenin, quercetin-3-rutinoside, kaempferol, isorhamnetin, etc.)	Peel	Quercetin-3-rhamnoside 11.66 mg/g, myricetin 12.10 mg/g in white-fleshed peel; quercetin-3-rutinoside predominant in red-skinned red-fleshed peel; catechin, epigallocatechin, vitexin, rutin, luteolin, apigenin detected Total flavonoids 5.72 mg RE/g DW	(21, 23–25)
	Flowers	Quercetin, kaempferol, isorhamnetin glycosides most abundant in flowers	(49)
	Seeds	Quercetin, kaempferol, isorhamnetin glycosides abundant in seeds	(10, 49)
Pectic polysaccharides (RG-I type)	Peel	Account for 15–20% of peel dry weight; RG-I domain content up to 66.59 mol%; (Ara + Gal)/Rha = 1.25; side-chain structure of RG-I domain is critical for prebiotic activity	(26–30)
Polysaccharides (cellulose, hemicellulose, hot water-extracted)	Flesh	Similar structural characteristics in FT-IR and NMR spectra among cultivars, but monosaccharide compositions differ significantly	(37)
	Stems	Cellulose and hemicellulose forms; significant differences in monosaccharide composition, molecular weight, and surface morphology among cultivars; hot water-extracted stem polysaccharides show superior antioxidant and hypoglycemic activities	(51)
Flower polysaccharides (FHRP-1, FHRP-2, FHRP-3)	Flowers	Three fractions obtained through water extraction and chromatographic purification; significantly enhanced antioxidant and immunomodulatory activities at 200–800 μg/mL	(53)
Unsaturated fatty acids (linoleic acid, oleic acid, palmitic acid)	Seeds	152 lipid species identified including glycerides and phospholipids; Linoleic acid 42.78%; oleic acid 27.29%; palmitic acid 16.66%; total unsaturated fatty acids 75.43%; saturated fatty acids 24.57%	(46, 47)
Triterpenoids (lupeol)	Stems	Lupeol isolated from stems	(52)
Galloylglucose derivatives (1-galloylglucose, 3-galloylglucose)	Seeds	Identified in seed extracts	(50)
Amino acids (ornithine, asparagine, glutamine, indole-3-lactic acid)	Flesh	Flesh rich in 18 amino acids (8 essential); red-fleshed pitaya particularly abundant in ornithine, asparagine, glutamine, and indole-3-lactic acid	(5, 38, 39)
Vitamin C	Flseh	~6 mg/100 g FW in both red- and white-fleshed; up to 8.92 ± 0.13 mg/g FW in specific cultivar; 22.291 mg/100 g in dried flesh, continuous accumulation during ripening	(5, 41–43)
Minerals and dietary fiber (potassium, ash, dietary fiber)	Peel	Potassium 4.43 g/100 g; ash 11.63 g/100 g; dietary fiber 56.56 g/100 g	(23)

### 2.1 Chemical composition of pitaya peel

The peel accounts for approximately 22% of the total pitaya fruit weight and is conventionally regarded as waste; however, it is chemically rich and has considerable utilization value.

#### 2.1.1 Polyphenols

Polyphenolic compounds were extracted from the peels of both red-skinned and white-skinned pitaya varieties using multiple extraction methods, including acid, alkaline, and composite enzymatic hydrolyses. The predominant free polyphenols in red-fleshed pitaya peel were identified as chlorogenic, caffeic, ferulic, and p-coumaric acids, whereas those in white-fleshed pitaya peel were chlorogenic acid, caffeic acid, rutin, and isoquercitrin (19). Bound polyphenols were extracted from pitaya peel using an ultrasound-alkali method, and the optimal yield of bound polyphenols was determined to be approximately 2.38–2.45 mg GAE/g DW (20).

#### 2.1.2 Flavonoids

Flavonoid compounds are also important constituents of pitaya peel (21). During the ripening process of three pitaya cultivars, the total phenolic and flavonoid contents, together with antioxidant activity, were significantly higher in the peel than in the flesh (22). Quercetin-3-rhamnoside (11.66 mg/g) and myricetin (12.10 mg/g) were detected as the principal flavonoid components in white-fleshed pitaya peel (23). Quercetin-3-rutinoside was identified as the predominant flavonoid common to both peel and flesh in the red-skinned, red-fleshed variety (24). UPLC analysis revealed the presence of multiple flavonoids in the peel extracts, including catechin, epigallocatechin, vitexin, rutin, luteolin, and apigenin (25). The 70% ethanol extract exhibited the strongest comprehensive antioxidant capacity, attributable to its highest total phenolic and total flavonoid contents (25).

#### 2.1.3 Polysaccharides

Polysaccharides in the peel predominantly occur in the form of pectin, constituting 15–20% of the dry weight. They possess thickening and gelling properties, rendering them suitable for use as stabilizers, thickeners, and gelling agents in food processing (26). Pectin obtained via citric acid extraction exhibited the highest rhamnogalacturonan-I (RG-I) domain content (66.59 mol%) and a more abundant neutral sugar side-chain structure ((Ara + Gal)/Rha = 1.25) (27). Structural characterization further indicated that the side-chain structure of the RG-I domain may be a critical factor influencing the prebiotic activity of pectin (28–30).

#### 2.1.4 Pigments

Chemical analysis revealed that the peel extracts contained high concentrations of betacyanin monomers, including betanin, isobetanin, phyllocactin, and isophyllocactin (31). LC–MS/MS analysis confirmed that isobetanin, phyllocactin, and isophyllocactin were the principal betalain components in red-fleshed pitaya, with phyllocactin being the predominant constituent. Derivatives such as 4’-O-malonylbetanin have also been detected (32). The betacyanin content in the dried peel powder was determined to be 9.65 mg/100 g (23). In red-fleshed pitaya peel extracts, betalains reached 35.12 mg/g and anthocyanins 19.21 mg/g, both of which were significantly higher than the corresponding contents in the flesh (betacyanin 30.15 mg/g, anthocyanins 15.16 mg/g); notably, betanin, as the principal betacyanin component, attained a content of 9.44 mg/g in the peel (33). As the principal functional pigment, betacyanin has been successfully extracted and applied in food nanofiber encapsulation technology (34). Some studies have detected anthocyanins (e.g., cyanidin-3-glucoside and delphinidin-3-glucoside) in red-fleshed pitaya flesh, whereas they were absent in white-fleshed varieties (21). Integrated metabolomic and transcriptomic analyses suggest that the anthocyanin biosynthetic pathway is differentially regulated in red-fleshed flesh, potentially contributing to color formation in conjunction with betalains (35); however, the specific contribution of this pathway remains to be further validated.

Furthermore, the potassium content in the peel powder reached 4.43 g/100 g, with ash (11.63 g/100 g) and dietary fiber (56.56 g/100 g) being significantly higher than those in the flesh powder (23).

### 2.2 Chemical composition of pitaya flesh

The flesh constitutes the primary edible portion of pitaya, and its chemical composition varies among cultivars. Red-fleshed pitaya is characterized by high betalain content, whereas white-fleshed pitaya is rich in polysaccharides.

#### 2.2.1 Polyphenols

Multiple phenolic compounds have been detected in white-fleshed pitaya flesh (10). Phenolic substances were extracted from both the peel and flesh of white-fleshed pitaya using microwave-assisted extraction, and subsequent HPLC-DAD analysis further clarified that the principal phenolic components in the flesh included chlorogenic acid, caffeic acid, rutin, luteolin-7-glucoside, and quercetin, whereas chlorogenic acid and quercetin predominated in the peel (36).

#### 2.2.2 Polysaccharides

Polysaccharides from the flesh of different pitaya cultivars exhibited similar structural characteristics in FT-IR and NMR spectra. However, their monosaccharide compositions differed significantly (37). These structural variations directly influence antioxidant activity. In contrast, the shared structural features exhibited relatively stable anti-inflammatory activity.

#### 2.2.3 Amino acids

Amino acids are the fundamental building blocks of proteins. The flesh is rich in 18 amino acids, of which 8 are essential (5). Red-fleshed pitaya is particularly rich in ornithine, asparagine, glutamine, and indole-3-lactic acid. These amino acids participate in carbohydrate metabolism, amino acid synthesis, and metabolic pathways (38). Furthermore, active components, such as phenolic acids and flavonoids in pitaya, may synergistically enhance amino acid bioavailability (39).

#### 2.2.4 Betalains

The betalain content in red-fleshed cultivars was significantly higher than that in white-fleshed cultivars (18). Ultrasound-assisted extraction of red-fleshed pitaya flesh revealed that the betacyanin concentration in the extract reached 15.18–20.03 mg/100 mL, with betaxanthins at 6.39–8.31 mg/100 mL (40). The flesh extract demonstrated high safety and low cytotoxicity, rendering it suitable as a source of antioxidant constituents for daily dietary intake (33).

#### 2.2.5 Vitamin C

Vitamin C in pitaya flesh constitutes an important nutritional and bioactive component; its presence has been confirmed, and its relevant characteristics have been investigated in multiple studies. The vitamin C content in both red-fleshed and white-fleshed pitaya flesh is approximately 6 mg/100 g FW (5); further comparative analysis revealed significant inter-cultivar differences and a continuous accumulation trend during fruit ripening (41). The vitamin C content in a particular cultivated variety reached as high as 8.92 ± 0.13 mg/g FW (42). The vitamin C content in dried flesh was 22.291 mg/100 g; however, this figure represents a processed product and does not reflect the original level in fresh flesh (43). Peracetic acid treatment significantly increased the vitamin C content in the flesh and maintained its antioxidant capacity (44). When high hydrostatic pressure treatment was applied to pitaya-pineapple blended beverages, the retention rate of vitamin C in the system was elevated to 64% (45).

### 2.3 Chemical composition of pitaya seeds

Although pitaya seeds constitute only 1–2% of the total fruit weight, they are abundant in unsaturated fatty acids and phytosterols (46).

#### 2.3.1 Unsaturated fatty acids

Pitaya seeds contain substantial quantities of unsaturated fatty acids, which exert hypolipidemic effects and contribute to the prevention of cardiovascular diseases. Analysis of the lipid composition of pitaya seed oil revealed the following proportional contents of different fatty acids: linoleic acid, 42.78%; oleic acid, 27.29%; and palmitic acid, 16.66%. The total unsaturated fatty acid content reached 75.43% (polyunsaturated fatty acids 43.07%, monounsaturated fatty acids 32.36%), whereas the saturated fatty acid content was 24.57% (47).

#### 2.3.2 Polyphenols and flavonoids

Chemical analysis revealed that the polyphenol and flavonoid contents in the seeds of white-fleshed pitaya were significantly higher than those in the flesh (10). The seeds of white-fleshed pitaya exhibited a relatively high content of polyphenolic compounds, with a broad range of total phenolic content (52–144 mg/g) and a mean value of 100.83 mg/g (48). The seeds are rich in flavonoids, including glycoside derivatives such as quercetin, kaempferol, and isorhamnetin (49).

Furthermore, galloylglucose derivatives (e.g., 1-galloylglucose and 3-galloylglucose) were identified in seed extracts, and these constituents are closely associated with antioxidant and anti-glycation activities (50).

### 2.4 Chemical composition in pitaya stems

The chemical components of pitaya stems primarily include polysaccharides, flavonoids, and triterpenoids.

#### 2.4.1 Polysaccharides

These exist mainly in the form of cellulose and hemicellulose. NMR and FT-IR analyses of polysaccharides from pitaya stems indicated that they shared similar glycosyl types; however, polysaccharides from different cultivars exhibited significant differences in monosaccharide composition, molecular weight, and surface morphology (37). Polysaccharides extracted with hot water have demonstrated superior antioxidant and hypoglycemic activities (51).

#### 2.4.2 Triterpenoids

Lupeol, isolated from the stems of red-fleshed pitaya, is a triterpenoid compound with immunomodulatory effects (52).

### 2.5 Chemical composition in pitaya flowers

Pitaya flowers, which have both medicinal and edible properties, contain various bioactive chemical components.

#### 2.5.1 Polysaccharides

Three polysaccharide fractions (FHRP-1, FHRP-2, and FHRP-3) were obtained through water extraction and chromatographic purification, which significantly enhanced antioxidant and immunomodulatory activities at concentrations ranging from 200 to 800 μg/mL (53).

#### 2.5.2 Flavonoids

Twelve flavonoid compounds were identified in pitaya flowers, among which glycoside derivatives of three flavonols—quercetin, kaempferol, and isorhamnetin—were the most abundant (49).

Furthermore, a comparative phytochemical analysis of different tissue parts of white-fleshed pitaya indicated that the floral parts might also be rich in secondary metabolites, exhibiting considerable potential for bioactivity (10). Pitaya flower extracts may influence the pathological progression of chronic allergic respiratory diseases, such as asthma, by modulating the gut–lung axis, thereby offering novel insights into natural intervention strategies for related disorders (54).

## 3 Biological activities and mechanisms of action

### 3.1 Antioxidant activity

Oxidative stress refers to cellular damage resulting from an imbalance between the production and elimination of free radicals *in vivo* (55). Organisms have evolved a complex antioxidant defense system to counteract such damage, in which antioxidant-active components play a pivotal role (56, 57). Pitaya is rich in various antioxidant-active components, including polyphenols, flavonoids, triterpenoids, and betacyanins (20). These components can further strengthen the antioxidant defense system by directly scavenging ROS and modulating antioxidant enzyme activity (58). They react directly with free radicals to reduce their reactivity, thereby attenuating oxidative damage to biological macromolecules and contributing to the delay of aging and prevention of oxidative stress-related diseases, such as cardiovascular diseases and cancer (59).

*In vitro* studies have demonstrated that polyphenolic compounds from pitaya exhibit favorable scavenging capacities against DPPH radical, OH radical, and ABTS radical, and enhanced FRAP (38, 60–62). Notably, the antioxidant properties varied among different plant parts: the DPPH radical-scavenging activity of the peel was lower than that of the flesh; however, under hydrogen peroxide-induced oxidative stress conditions, peel extracts demonstrated superior cytoprotective effects, suggesting potential functional complementarity in antioxidant mechanisms across different parts (33). Pitaya digestion products alleviate oxidative stress-induced damage to gastric, intestinal, and hepatic cells by reducing intracellular ROS levels, and this cytoprotective effect is closely associated with the chemical antioxidant capacity of its polyphenols (63). In animal experiments, extracts from red-fleshed pitaya reduced malondialdehyde (MDA) levels in hepatic tissue and elevated total antioxidant capacity (T-AOC) and total superoxide dismutase (T-SOD) activity (64). In diabetic rat models, pitaya consumption significantly decreased MDA levels (65). In a copper-induced neurotoxicity model in adult zebrafish, pitaya extract attenuated oxidative stress and ameliorated impaired cholinergic nervous system function (66). Similar improvements in oxidative damage markers have been observed in exercise animal models and aflatoxin B1 (AFB1)-induced rat oxidative injury models (67, 68).

Several studies have reported alterations in proteins associated with the Nrf2/HO-1 signaling pathway (upregulation of Nrf2 and HO-1 and downregulation of Keap1) and CYP2E1 expression, as well as modulation of the Nrf2/TXNIP/NLRP3 inflammasome pathway (68–71). However, these findings provide correlational evidence and do not establish direct causality. The current study has several notable limitations. First, substantial heterogeneity in extract types, dosages, and experimental models across studies precludes the direct comparison of findings. Second, the extent to which *in vitro* free radical scavenging capacity translates into *in vivo* antioxidant efficacy remains poorly understood. Third, reliance on isolated antioxidant biomarkers is inadequate for predicting multifaceted biological outcomes. Most critically, well-designed human clinical trials to substantiate the practical antioxidant benefits are currently lacking.

### 3.2 Anti-inflammatory activity

Inflammation is a defensive physiological response triggered by the immune system in response to pathogen invasion or tissue injury. Under normal regulatory conditions, this response facilitates pathogen clearance and promotes tissue repair (72). However, when the inflammatory reaction becomes excessive or persists chronically, it may evolve into chronic inflammation, thereby contributing to the initiation and progression of various diseases, including cancer, type II diabetes, and cardiovascular diseases. Sustained inflammatory responses may lead to cellular damage, tissue dysfunction, and disease development (73, 74). Pitaya contains multiple anti-inflammatory active components, such as polyphenols and flavonoids (75, 76), and its anti-inflammatory activity is primarily achieved by suppressing inflammatory signaling pathways, effectively inhibiting the release of inflammatory mediators and cytokines generated during the inflammatory process (77).

The anthocyanin components (principally cyanidin-3-glucoside) present in the red flesh, red peel, and white peel of pitaya exert anti-inflammatory activity maybe through a multi-level synergistic mechanism: at the downstream level, they directly inhibit iNOS and COX-2 protein expression, thereby reducing the production of inflammatory mediators such as NO and PGE₂; at the intermediate level, they block NF-κB nuclear translocation and suppress the AP-1 signaling pathway, downregulating pro-inflammatory transcriptional activity; at the upstream level, they activate the Nrf2/ARE antioxidant defense pathway, enhancing the expression of endogenous antioxidant enzymes, which indirectly attenuates oxidative stress-driven chronic inflammation (15, 63, 78–80). Furthermore, in diabetic complications, pitaya components can inhibit the release of inflammatory factors by modulating the NLRP3 inflammasome, thereby alleviating tissue damage (70). These mechanisms operate in concert, enabling pitaya to effectively mitigate oxidative stress-induced inflammation (81). Similarly, flavonoid glycosides such as quercetin and kaempferol extracted from pitaya flowers can downregulate COX-2 protein expression and reduce the production of inflammatory mediators, including prostaglandins, by inhibiting the NF-κB and MAPK pathways (49). Gold nanoparticles (Au-NPs) ith multifaceted biological activities were successfully synthesized via a green synthesis method utilizing the flesh and seed oil extracts of red-fleshed pitaya as reducing and stabilizing agents; the inhibitory effects of Au-NPs on COX-1 and COX-2 enzymes indicated their anti-inflammatory potential (82). Additionally, fermentation or thermal processing of red-fleshed pitaya converts its polyphenols into small-molecule derivatives, such as caffeic acid, further enhancing their bioactivity (83).

In summary, pitaya effectively modulates key inflammatory mediators, including ROS/RNS, COX-2, and iNOS, through the synergistic antioxidant and anti-inflammatory effects of its polyphenolic components, providing a scientific foundation for the development of natural anti-inflammatory food products. However, current research on the anti-inflammatory activity of pitaya remains predominantly confined to *in vitro* cellular experiments, with limited data from animal models. However, the existing evidence regarding anti-inflammatory pathways is insufficient to support definitive conclusions. Studies on the enhancement of activity through fermentation or thermal processing lack standardized process parameters, raising concerns regarding reproducibility. Moreover, human clinical trial data are required for further validation of these findings.

### 3.3 Antimicrobial activities

Pitaya exhibits broad-spectrum antimicrobial activity, with extracts from its peel and flesh demonstrating inhibitory effects against various pathogenic bacteria and fungi.

*In vitro* studies have demonstrated that aqueous extracts of pitaya peel exhibit inhibitory activity against Gram-negative bacteria, including *Escherichia coli*, *Salmonella*, and *Pseudomonas aeruginosa*; Gram-positive bacteria, such as *Staphylococcus aureus* and its *methicillin-resistant strain*; and the *fungus Candida albicans* (84). Polyphenols and betacyanins present in red-fleshed pitaya extracts effectively inhibit the growth of aerobic, anaerobic, and proteolytic bacteria, which is attributed to their capacity to disrupt microbial cell membrane integrity and interfere with energy metabolism (85). The pitaya-gelatin films exhibited preservative effects owing to the presence of these components. Furthermore, pitaya extracts have demonstrated inhibitory activity against pathogenic fungi such as *Rhizoctonia solani*, *Aspergillus flavus*, *Botrytis cinerea*, *Fusarium oxysporum*, and *Cladosporium herbarum* (86). Lactic acid fermentation of pitaya juice significantly elevated the total phenolic and flavonoid contents while enhancing α-glucosidase and lipase inhibitory activities, thereby indirectly affecting microbial carbohydrate metabolic pathways (38, 87). Fermentation also generates substantial quantities of low-molecular-weight phenolic derivatives (e.g., cinnamic acid and 6-shogaol), further broadening the antimicrobial spectrum (83). The combined application of pitaya extracts with vanillin inhibited postharvest soft rot pathogens, with the mechanism involving the disruption of fungal cell wall synthesis and induction of oxidative stress (88). Nano-selenium biofortified pitaya activated the phenylpropanoid and betacyanin biosynthetic pathways, enhanced antioxidant enzyme (SOD and CAT) activity, and indirectly augmented its antimicrobial capacity (89).

Current research on antimicrobial activity consists almost exclusively of *in vitro* experiments, with a paucity of animal infection models and clinical data. Moreover, the proposed mechanisms of action remain largely speculative and have not been rigorously validated.

### 3.4 Lipid-regulatory activity

Hyperlipidemia is a metabolic disorder characterized by abnormally elevated blood lipid levels and is a core risk factor for cardiovascular disease, which can precipitate myocardial infarction and stroke and exacerbate vascular damage by promoting intracellular lipid peroxidation (90, 91). Clinically, it manifests as elevated levels of total cholesterol (TC), triglycerides (TG), and low-density lipoprotein cholesterol (LDL-C) in the peripheral blood (92). Red-fleshed pitaya is rich in various bioactive components, including betacyanins, polyphenols, flavonoids, and phenolic acids, and both *in vitro* and animal studies have demonstrated its potential lipid-regulatory activity (12, 21).

*In vitro* experiments have shown that red-fleshed pitaya peel extracts exhibit no cytotoxicity in cellular assays (33). Although the specific molecular mechanisms remain to be elucidated, bioactive components may influence lipid metabolism through multiple pathways, demonstrating the capacity to ameliorate dyslipidemia in *in vitro* evaluations (93). Additionally, the fatty acid composition of pitaya seed oil requires further investigation. Its principal constituents are linoleic acid (42.78%), oleic acid (27.29%), and palmitic acid (16.66%), comprising 152 lipid species, including glycerides and phospholipids. This fatty acid profile is considered beneficial for cardiovascular health and may indirectly support lipid regulation, although this conclusion remains a compositional inference rather than an experimentally derived finding (47). In animal experiments, fermented red-fleshed pitaya demonstrated more definitive hypolipidemic effects. Studies have revealed that red-fleshed pitaya fermented with *Bifidobacterium breve* or *Lactobacillus casei* improves glycemic control and lipid metabolism in diabetic mouse models (87). Furthermore, metabolomic analysis indicated that fermented red-fleshed pitaya juice significantly elevated total phenolic, flavonoid, and anthocyanin contents, enhanced lipase inhibitory activity, and modulated non-volatile components associated with lipid and carbohydrate metabolism (38). Additional experiments have demonstrated that pitaya extracts reduce serum TG and LDL-C levels (94), reverse hepatic energy balance disturbances induced by high-carbohydrate and high-fat diets and regulate inflammatory factor expression (79). Dietary administration of red-fleshed pitaya peel extract also ameliorated the lipid profile in mice (95).

However, the specific molecular mechanisms underlying lipid regulatory activity remain unclear. Although pitaya consumption may contribute to the improvement of lipid profiles, no studies have investigated its cardioprotective effects in human trials, particularly at nutritionally achievable doses. Although the hypolipidemic potential of pitaya has been proposed, it has not yet been validated through clinical investigations (96).

### 3.5 Hypoglycemic effects

Hyperglycemia refers to a pathological state characterized by abnormally elevated blood glucose concentrations, including impaired fasting glucose and impaired glucose tolerance, and is one of the principal features of diabetes mellitus (97).

First, pitaya has a relatively high dietary fiber content, which can attenuate intestinal glucose absorption through physical barrier effects (5). Second, microencapsulated pitaya flesh extracts demonstrated significant antioxidant activity in copper-induced toxicity models of nematodes and zebrafish, thereby alleviating oxidative damage closely associated with insulin resistance (66, 98). Furthermore, in animal experiments, β-cyanins from pitaya extracts ameliorated insulin resistance in high-fat diet-fed mice and modulated the adipose metabolic pathway FGF21 (18, 99). The combined administration of pitaya with metformin reduced the HOMA-IR index in diabetic models, suggesting its capacity to synergistically enhance peripheral tissue glucose uptake (100, 101). In streptozotocin-nicotinamide (STZ-NA)-induced hyperglycemic rat models, pitaya stem and rhizome extracts exhibited acute hypoglycemic effects, which may be attributed to their inhibition of glucose-6-phosphatase and fructose-1,6-bisphosphatase activities, thereby reducing hepatic glucose output and contributing to the regulation of fasting blood glucose levels (100). Pitaya extracts also demonstrated favorable hypoglycemic effects in diabetic rat models, significantly lowering blood glucose levels while concurrently ameliorating dyslipidemia, elevating insulin levels, and increasing high-density lipoprotein cholesterol levels, suggesting comprehensive metabolic regulatory effects (102, 103). More in-depth mechanistic studies revealed that pitaya leaf extracts not only significantly reduced fasting blood glucose and glycated hemoglobin levels, but also improved lipid profiles, increased hepatic glycogen reserves, and enhanced antioxidant enzyme activity. These extracts upregulated the gene expression of glucose transporters GLUT-2 and GLUT-4, as well as hexokinase, indicating that they exert hypoglycemic effects by promoting glucose uptake and utilization and improving insulin sensitivity (104).

In clinical studies, pitaya has been shown to reduce fasting blood glucose levels in patients with prediabetes; however, its hypoglycemic efficacy in patients with type 2 diabetes mellitus remains inconclusive, and further rigorously designed clinical investigations are warranted to confirm its activity potency, effective dosage, and long-term benefits (105).

### 3.6 Hepatoprotective effects

Multiple bioactive components of pitaya have been shown to exert significant hepatoprotective effects. They can attenuate the severity of hepatic injury, promote hepatocyte repair and regeneration, and reduce the risk of liver disease.

In a CCl₄-induced acute hepatic injury rat model, oral administration of 300 mg/kg methanolic pitaya extract exhibited hepatoprotective effects, with certain parameters comparable to those of the classical hepatoprotective drug silymarin (106). In the C57BL/6 mouse ALD model, red-fleshed pitaya peel extract ameliorated alcohol-induced hepatic injury and hepatic lipid accumulation by modulating lipid metabolism, reducing oxidative stress, and attenuating inflammatory responses (69). Red-fleshed pitaya juice demonstrated potential hepatoprotective effects in a high-fat and high-sugar diet-induced metabolic syndrome rat model; however, changes in key gene expression failed to reach statistical significance, inflammatory factor alterations were contradictory in direction, and classical liver function indices and histological validation were lacking (79). In an AFB1-induced rat hepatic injury model, betacyanins, polyphenols, and flavonoids from red-fleshed pitaya juice significantly alleviated aflatoxin-induced hepatocellular damage by activating the Nrf2 antioxidant pathway, inhibiting lipid peroxidation, and restoring hepatic antioxidant enzyme activity, thereby exhibiting favorable hepatoprotective activity (68). Additionally, pitaya juice concentrate conferred a certain degree of protective effect against cisplatin (CP)-induced nephrotoxicity (107).

Research on the hepatoprotective activity of pitaya has substantial limitations. Studies remain at the animal model stage, and their efficacy in humans remains unknown. Furthermore, the absence of standardized dosages and divergence in pathological mechanisms across different models render the integration of these findings problematic.

### 3.7 Prebiotic effects

Pectin and other dietary fiber components abundant in pitaya have been demonstrated to possess significant prebiotic properties that modulate intestinal microecological balance through multiple mechanisms (27). Their effects are primarily manifested in the promotion of beneficial bacterial proliferation and optimization of the microbial community structure (108).

In *in vitro* fermentation and simulated digestion experiments, pitaya flesh, rich in moisture (85.83 g/100 g), carbohydrates (11.65 g/100 g), and dietary fiber (2.49 g/100 g), served as a growth substrate for probiotics. Following fermentation with the probiotic strains *Lacticaseibacillus paracasei subsp. paracasei* F-19 (F-19) and *Bifidobacterium animalis subsp. lactis* BB-12 (BB-12), F-19 exhibited a stronger acidification capacity (pH reduced to 3.6) and specifically produced 2-phenylethanol, a flavor compound with a rose aroma. Both strains delayed betacyanin degradation, and the fermented products showed an initial increase in total phenolic content, with a slight overall decrease in antioxidant activity. Furthermore, the red-fleshed pitaya matrix significantly enhanced the survival rate of F-19 during simulated gastrointestinal digestion, with viable bacterial counts remaining high (>7.97 log CFU/mL) after simulated gastric and intestinal fluid treatment (109). After 48-h fermentation of red-fleshed pitaya flesh by *Lactobacillus acidophilus* LA-05 and *Bifidobacterium animalis* ssp. *lactis* BB-12 at 30 °C, *in vitro* simulated gastrointestinal digestion and dialysis membrane-simulated intestinal barrier assessment of polyphenol bioaccessibility revealed that fermentation enhanced the bioaccessibility of specific polyphenols (LA-05 increased catechin by 372%, epigallocatechin gallate by 181%, and procyanidin B2 by 311%; BB-12 increased catechin and procyanidin B2 by 43 and 33%, respectively). The non-dialyzable fractions (colon-available portions) showed increased polyphenol content and elevated ORAC values (110). Following 48-h fermentation of red-fleshed pitaya flesh by *Lacticaseibacillus casei* and *Bifidobacterium breve* at 37 °C, *in vitro* simulated gastrointestinal digestion and human fecal microbiota *in vitro* colonic fermentation assessments demonstrated that the high-molecular-weight oligosaccharides produced through fermentation selectively promoted the proliferation of beneficial intestinal bacteria. 16S rRNA sequencing revealed increased abundance of probiotics, including *Lactobacillus*, *Bifidobacterium*, and *Faecalibacterium prausnitzii*, alongside reduced abundance of the potential pathogen *Sutterella* (111). In animal experiments, pitaya pomace polysaccharides extracted from pitaya processing by-products were administered to high-fat diet-induced obese C57BL/6 mouse models, revealing definitive prebiotic effects. 16S rRNA sequencing demonstrated the restoration of beneficial bacteria, including *Romboutsia*, *Lachnospiraceae*_NK4A136_group, *Coriobacteriaceae*_UCG-002, and *Blautia*, reduction of the inflammation-associated bacterium *Mucor sphaericus,* reversal of high-fat diet-induced F/B imbalance and decreased *Bifidobacteriaceae* abundance, and promotion of SCFAs production through intestinal microbiota metabolism (112).

Currently, the prebiotic properties of pitaya are based exclusively on *in vitro* experiments and a single animal model, with substantial variations in fermentation conditions, strain combinations, and assessment methods across studies, making it difficult to integrate the results. The prebiotic potential remains supported solely by low-grade, fragmented preclinical evidence, and its human applicability and safety await confirmation through rigorously designed clinical trials (Table 2).

**Table 2:** Biological activities of phytochemical constituents from pitaya (*Selenicereus* spp.).

Biological activity	Active compound	Extraction method	Experimental model	Molecular mechanism	Observed indicators	References
Antioxidant activity	Betacyanins (betanin and phyllocactin) from red-fleshed peel	Aqueous extraction with ethanol precipitation, macroporous resin purification	Copper-induced toxicity model in *Caenorhabditis elegans*	Direct ROS scavenging; activation of SOD/CAT antioxidant enzymes	Survival rate, ROS levels, lipofuscin accumulation, SOD activity, CAT activity	(98)
	Betacyanin extract from red-fleshed peel	Ultrasound-assisted aqueous extraction, AB-8 resin purification	Copper-induced toxicity model in adult zebrafish	Mitigation of oxidative stress; protection of cholinergic neurons; reduction of lipid peroxidation	Amelioration of behavioral abnormalities, cell death markers, lipid peroxidation, restoration of cholinergic nervous system function	(66)
	Polyphenols from red-fleshed peel	Ultrasound-assisted ethanol extraction, rotary evaporation concentration	Acute swimming exercise rat model	Enhancement of endogenous antioxidant defense system; reduction of exercise-induced oxidative damage	Serum lactate, creatine kinase, MDA, GSH, SOD, CAT	(67)
	Red-fleshed pitaya juice (betacyanins, polyphenols, flavonoids)	Juice preparation, betacyanin-rich fraction	AFB1-induced rat hepatic injury model	Activation of Nrf2/HO-1 pathway; inhibition of CYP2E1 expression; modulation of Nrf2/TXNIP/NLRP3 inflammasome pathway	Serum ALP, hepatic TBARS, SOD, CAT, GPx, GSH, MDA, Nrf2, HO-1, Keap1, CYP2E1 expression	(68)
	Red-fleshed pitaya peel extract	Ethanol extraction	Alcoholic liver disease C57BL/6 mouse model	Modulation of lipid metabolism; reduction of oxidative stress; attenuation of inflammatory responses	Hepatic MDA levels, T-AOC, T-SOD activity, lipid accumulation, liver function markers	(69)
	Red-fleshed pitaya extract	Aqueous extraction	Diabetic rat model	Reduction of oxidative stress; enhancement of antioxidant capacity	MDA levels, T-AOC, T-SOD, blood glucose, lipid profile	(64, 65)
	Pitaya digestion products (polyphenols)	*In vitro* gastrointestinal digestion	Oxidative stress-induced gastric, intestinal, and hepatic cell damage	Reduction of intracellular ROS levels; chemical antioxidant capacity of polyphenols	Intracellular ROS, cell viability, cytoprotective effects	(63)
Anti-inflammatory activity	Anthocyanins (from red flesh, red peel, and white peel)	Ethanol extraction, purification	LPS-induced RAW264.7 macrophages; Inflammatory cell models	Inhibition of iNOS and COX-2 protein expression; blockade of NF-κB nuclear translocation; suppression of AP-1 signaling; activation of Nrf2/ARE pathway	NO production, PGE₂ levels, iNOS, COX-2, NF-κB, AP-1, Nrf2, ARE expression	(15, 63, 78–80)
	Flavonoid glycosides from pitaya flowers	Hot-air and vacuum drying, ethanol extraction	Inflammatory cell models	Downregulation of COX-2 protein expression; inhibition of NF-κB and MAPK pathways; reduction of prostaglandin production	COX-2 expression, prostaglandin levels, NF-κB, MAPK pathway markers	(49)
	Au-NPs synthesized from red-fleshed pitaya flesh and seed oil extracts	Green synthesis using pitaya extracts as reducing and stabilizing agents	*In vitro* enzyme inhibition assays	Inhibition of COX-1 and COX-2 enzymes	COX-1, COX-2 inhibitory activity	(82)
	Polyphenols (caffeic acid, cinnamic acid, 6-shogaol derivatives)	Probiotic fermentation; thermal processing	*In vitro* cellular models	Phenolic remodeling into small-molecule derivatives; enhanced bioactivity through biotransformation	Polyphenol profiles, anti-inflammatory mediator levels	(83)
Antimicrobial activities	Aqueous extracts of pitaya peel	Aqueous extraction	*In vitro* bacterial and fungal cultures	Inhibition of Gram-negative and Gram-positive bacteria; disruption of microbial cell membrane integrity	Growth inhibition of *E. coli*, Salmonella, *P. aeruginosa*, *S. aureus* (including MRSA), *C. albicans*	(84)
	Polyphenols and betacyanins from red-fleshed pitaya extracts	Ethanol/aqueous extraction	*In vitro* microbial cultures; pitaya-gelatin film preservation tests	Disruption of microbial cell membrane integrity; interference with energy metabolism	Inhibition of aerobic, anaerobic, and proteolytic bacteria; film preservative effects	(85)
	Pitaya extracts	Solvent extraction	*In vitro* fungal cultures	Inhibition of fungal growth	Growth inhibition of Rhizoctonia solani, *A. flavus*, *B. cinerea*, F. oxysporum, *C. herbarum*	(86)
	Lactic acid fermented pitaya juice	Lactic acid fermentation; elevated total phenolic and flavonoid contents	*In vitro* microbial assays	Enhanced α-glucosidase and lipase inhibitory activities; affected microbial carbohydrate metabolic pathways; generation of cinnamic acid, 6-shogaol	Total phenolics, flavonoids, α-glucosidase inhibition, lipase inhibition, antimicrobial spectrum	(83, 87)
	Pitaya extracts combined with vanillin	Combined application	Postharvest soft rot pathogen inhibition	Disruption of fungal cell wall synthesis; induction of oxidative stress	Postharvest soft rot inhibition, fungal cell wall integrity, oxidative stress markers	(88)
	Nano-selenium biofortified pitaya	Nano-selenium biofortification during cultivation	Biofortified pitaya extracts	Activation of phenylpropanoid and betacyanin biosynthetic pathways; enhanced SOD and CAT activity	Phenylpropanoid pathway markers, betacyanin content, SOD, CAT activity	(89)
Lipid-regulatory activity	Red-fleshed pitaya peel extracts	Solvent extraction	*In vitro* cellular assays	Multiple pathways influencing lipid metabolism; amelioration of dyslipidemia	Cell viability, lipid metabolism markers	(33, 93)
	Fermented red-fleshed pitaya juice	Probiotic fermentation; untargeted metabolomics	*In vitro* and *in vivo* models	Elevated total phenolic, flavonoid, and anthocyanin contents; enhanced lipase inhibitory activity	Total phenolics, flavonoids, anthocyanins, lipase inhibition	(38)
	Fermented red-fleshed pitaya	Probiotic fermentation; metabolomic analysis	Diabetic mouse models	Improved glycemic control and lipid metabolism; enhanced lipase inhibitory activity; modulation of lipid and carbohydrate metabolism-associated non-volatile components	Blood glucose, lipid profile, lipase inhibition, metabolomic markers	(87)
	Pitaya extracts	Extract preparation	High-carbohydrate and high-fat diet-induced metabolic syndrome rat model	Reversal of hepatic energy balance disturbances; regulation of inflammatory factor expression	Serum TG, LDL-C levels, hepatic energy balance, inflammatory factors	(79, 94)
	Red-fleshed pitaya peel extracts	Dietary administration	High-fat diet-induced mice	Amelioration of lipid profile	Serum lipid profile, hepatic lipid markers	(95)
Hypoglycemic effects	Whole pitaya fruit (dietary fiber)	Whole fruit consumption	General metabolic studies	Physical barrier effects attenuating intestinal glucose absorption	Blood glucose levels, glucose absorption rate	(5)
	Microencapsulated pitaya flesh extracts	Microencapsulation	Copper-induced toxicity models in nematodes and zebrafish	Significant antioxidant activity; alleviation of oxidative damage associated with insulin resistance	Survival rate, ROS levels, oxidative damage markers, insulin resistance indicators	(66, 98)
	β-cyanins from pitaya extracts	Betacyanin extraction	High-fat diet-fed mice	Amelioration of insulin resistance; modulation of adipose metabolic pathway FGF21	Insulin resistance markers, FGF21 expression, blood glucose, lipid profile	(18, 99)
	Pitaya combined with metformin	Combined administration	Diabetic models	Reduced HOMA-IR index; enhanced peripheral tissue glucose uptake	HOMA-IR, blood glucose, insulin levels, glucose uptake	(100, 101)
	Stem and rhizome extracts of pitaya	Aqueous/methanolic extraction	STZ-NA-induced hyperglycemic rat models	Inhibition of glucose-6-phosphatase and fructose-1,6-bisphosphatase activities; reduction of hepatic glucose output	Fasting blood glucose, hepatic glucose output, glucose-6-phosphatase, fructose-1,6-bisphosphatase activities	(100)
	Pitaya extracts	Extract preparation	Diabetic rat models	Comprehensive metabolic regulatory effects	Blood glucose, dyslipidemia amelioration, insulin levels, HDL-C levels	(102, 103)
	Pitaya leaf extracts	Aqueous extraction	Diabetic animal models	Upregulation of GLUT-2 and GLUT-4 gene expression; upregulation of hexokinase; promotion of glucose uptake and utilization; improvement of insulin sensitivity	Fasting blood glucose, glycated hemoglobin, lipid profile, hepatic glycogen reserves, antioxidant enzyme activity, GLUT-2, GLUT-4, hexokinase expression	(104)
	Whole pitaya fruit	Clinical consumption	Human clinical studies (prediabetic patients)	Reduction of fasting blood glucose	Fasting blood glucose levels	(105)
Hepatoprotective effects	Methanolic pitaya extract	Methanolic extraction	CCl₄-induced acute hepatic injury rat model	Hepatoprotective effects comparable to silymarin	Liver function parameters, hepatic histology, comparable to silymarin	(106)
	Red-fleshed pitaya peel extracts	Ethanol extraction	C57BL/6 mouse ALD model	Modulation of lipid metabolism; reduction of oxidative stress; attenuation of inflammatory responses	Alcohol-induced hepatic injury, hepatic lipid accumulation, oxidative stress markers, inflammatory markers	(69)
	Red-fleshed pitaya juice	Juice preparation	High-fat and high-sugar diet-induced metabolic syndrome rat model	Potential hepatoprotective effects	Liver function indices	(79)
	Betacyanins, polyphenols, and flavonoids from red-fleshed pitaya juice	Juice preparation, component fractionation	AFB1-induced rat hepatic injury model	Activation of Nrf2 antioxidant pathway; inhibition of lipid peroxidation; restoration of hepatic antioxidant enzyme activity	Hepatocellular damage, lipid peroxidation, antioxidant enzyme activity, Nrf2 pathway markers	(68)
	Pitaya juice concentrate	Juice concentration	CP-induced nephrotoxicity model	Protective effect against cisplatin-induced nephrotoxicity	Nephrotoxicity markers, renal function parameters	(107)
Prebiotic effects	Pitaya flesh	Whole flesh as growth substrate	Simulated gastrointestinal digestion	Growth substrate for probiotics; delayed betacyanin degradation; enhanced probiotic survival during digestion	pH reduction, 2-phenylethanol production, betacyanin retention, viable bacterial counts	(109)
	Red-fleshed pitaya flesh polyphenols (fermented)	Fermentation at 30 °C	*In vitro* simulated gastrointestinal digestion and intestinal barrier assessment	Enhanced bioaccessibility of specific polyphenols; increased colon-available polyphenol content and ORAC values	Catechin, epigallocatechin gallate, procyanidin B2, ORAC values in non-dialyzable fractions	(110)
	Red-fleshed pitaya flesh oligosaccharides (fermented)	Fermentation at 37 °C	*In vitro* simulated digestion and human fecal microbiota colonic fermentation; 16S rRNA sequencing	High-molecular-weight oligosaccharides selectively promote beneficial bacteria proliferation; optimization of microbial community structure	Increased *Lactobacillus*, *Bifidobacterium*, *Faecalibacterium prausnitzii*; reduced *Sutterella* abundance	(111)
	Pitaya pomace polysaccharides	Polysaccharide extraction from pomace	High-fat diet-induced obese C57BL/6 mouse models	Restoration of beneficial bacteria; reversal of F/B imbalance; promotion of SCFA production through intestinal microbiota metabolism	Restored *Romboutsia*, *Lachnospiraceae*_NK4A136_group, *Coriobacteriaceae*_UCG-002, Blautia; reduced *Mucor sphaericus*; reversed F/B imbalance; increased SCFAs	(112)

## 4 Functional applications in the food industry

**Figure 2:**
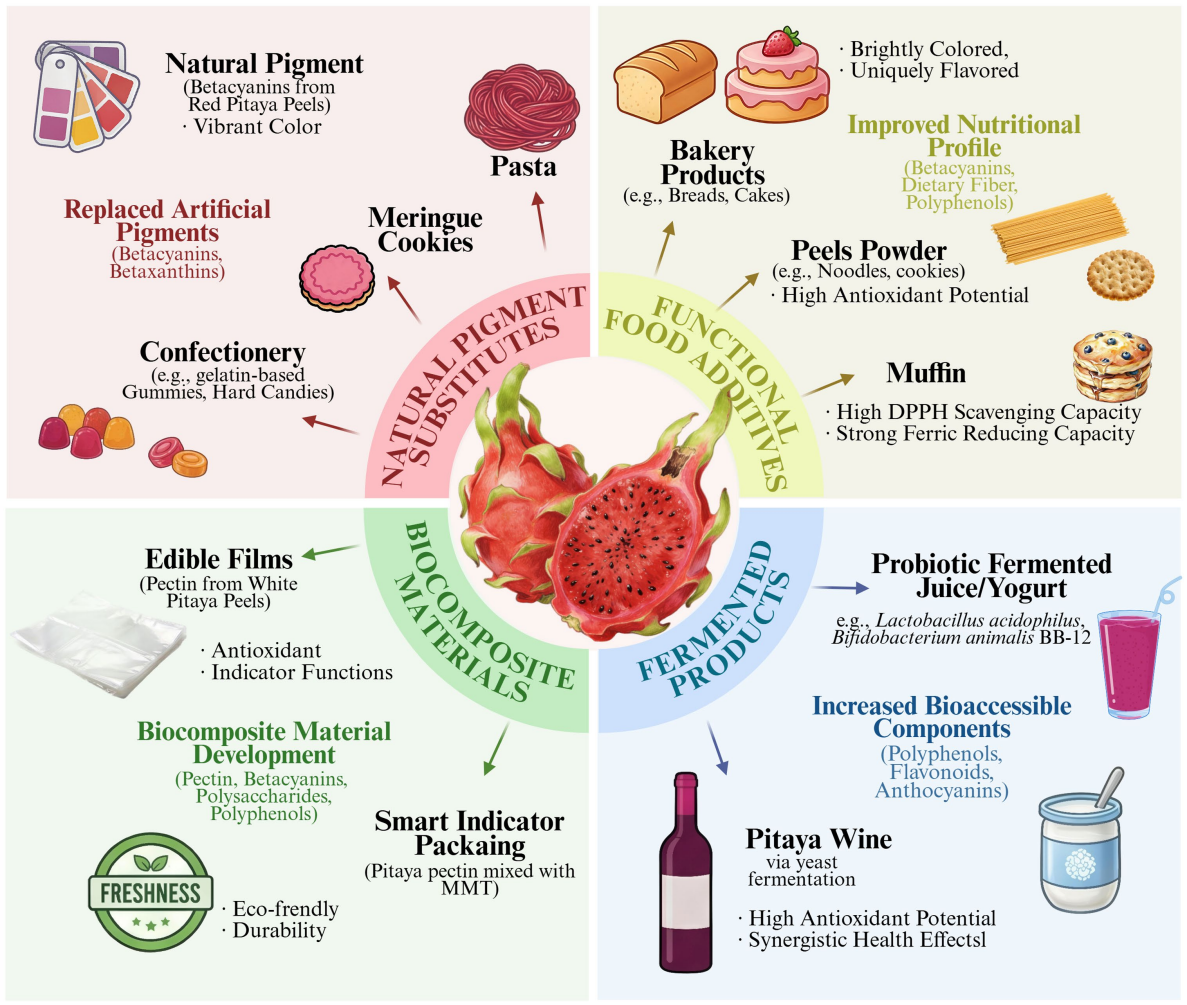
Functional food applications of pitaya (*Selenicereus* spp.). Natural pigments from peels replace artificial colorants in pasta, cookies, and confectionery. Bakery products enriched with peels powder or puree exhibit enhanced nutritional profiles and antioxidant capacity. Pectin-based edible films and smart packaging materials from by-products. Fermented products (probiotic juice/yogurt, wine) with increased bioaccessible polyphenols and synergistic health benefits (created in https://BioRender.com).

The abundant nutritional value and diverse bioactive components of pitaya confer extensive application prospects in the food sector; however, the majority of current research remains at the fundamental experimental stage, and industrial transformation faces considerable obstacles (Figure 2).

### 4.1 Fermented products

#### 4.1.1 Fruit juice

During the fermentation of red-fleshed pitaya juice, physicochemical properties, antioxidant activity, and volatile/non-volatile metabolites undergo changes, with total polyphenol, flavonoid, and anthocyanin contents increasing (38). Probiotic fermentation of pitaya juice significantly increased the total polyphenol, flavonoid, and anthocyanin contents, with the bioaccessible fraction exhibiting markedly higher antioxidant activity than unfermented juice, thereby providing a novel approach for enhancing its nutritional value and developing functional beverages (87, 110).

#### 4.1.2 Dairy products

Pitaya yogurt is a composite functional food that combines pitaya with yogurt. Pitaya is rich in polyphenolic substances and betacyanins, which possess biological activities, including antioxidant, anti-fatigue, and α-glucosidase inhibitory effects (111, 113). The incorporation of pitaya into yogurt not only improves the flavor and color of yogurt but also further enhances its functional characteristics through the fermentation process. Furthermore, polyphenols in pitaya have been demonstrated to exert anti-fatigue effects by modulating intestinal microbiota diversity (114). Yogurt, a fermented dairy product, contains viable probiotics that contribute to the maintenance of intestinal microecological balance (115). The combination of these two components forms a synbiotic system that may generate synergistic health effects (116). In summary, pitaya yogurt not only possesses the high nutrient density and probiotic properties of traditional yogurt but also integrates the phytochemical advantages of pitaya, representing a functional food with promising development prospects (117). However, the interaction between the acidic components of pitaya and milk proteins may lead to alterations in curd texture or phase separation, necessitating focused attention on product stability (118).

#### 4.1.3 Pitaya wine

Pitaya can also be utilized for the production of pitaya wine, which has a distinctive flavor and nutritional value (119). Pitaya wine also exhibits high total phenolic content, as well as superior DPPH scavenging capacity and FRAP, demonstrating excellent antioxidant potential (58). In addition to pure fruit fermentation, pitaya can be combined with honey to produce “Pitaya Melomel.” Pitaya pulp was added to diluted honey at concentrations ranging from 5 to 15%. The mixture was then fermented using *Saccharomyces cerevisiae*. This process resulted in a significant increase in total acidity. The incorporation of pitaya pulp enriched the final product with betalains (1.78–4.45 mg BE·L^−1^) and polyphenols (195.90–298.11 mg GAE·L^−1^). Furthermore, this fermentation approach promotes the formation of key aroma compounds, such as isoamyl acetate and nonanal. These compounds enhance the fruity and floral complexities of beverages. Overall, Pitaya Melomel represents a novel pathway for the valorization of pitaya in fermented beverages (120). Regarding brewing technology, studies have compared the effects of different yeast strains on the physicochemical properties of pitaya wine. The analytical results indicated that pitaya wines fermented with different yeast strains showed no significant differences in the content and types of volatile substances (121). Different yeast strains and fermentation temperatures influence the composition of volatile flavor compounds, and sequential inoculation methods can optimize aroma performance (122).

### 4.2 Functional food additives

Red-skinned pitaya is abundant in betacyanins, which possess favorable water solubility and vivid red-purple coloration, and have been identified as possessing potential for application as natural colorants in the food industry, particularly for pasta and baked products to replace artificial pigments (77). The incorporation of pitaya puree or fiber into dough at specific proportions significantly enhanced the redness value and overall color acceptability of the products, elevated dietary fiber and free phenolic acid contents, and strengthened the antioxidant capacity (123). Betacyanin-rich extracts from pitaya peels and flowers have been successfully applied as natural pigments in pasta and biscuits, exhibiting satisfactory color stability (124). Similarly, the addition of pitaya peel powder to noodles increased the betacyanin and flavonoid contents and enhanced the free radical scavenging capacity; however, attention must be paid to the potential impact of cooking processes on pigment stability and color (125). A study incorporating red-fleshed pitaya puree with seeds at specific proportions into muffins found that, after baking, the muffin surfaces presented dark coloration, while the interiors appeared yellow, with the original purple-pink hue essentially disappearing. Nevertheless, antioxidant performance evaluation demonstrated that muffins supplemented with pitaya waste material exhibited stronger DPPH radical scavenging capacity and ferric reducing power than control samples (126).

However, the muffin study explicitly revealed that, although the pink tone in the batter stage intensified with increasing puree proportion, the surfaces appeared dark and the interiors yellow after baking, with the original purple-pink hue essentially disappearing, indicating that the thermal instability of betacyanins constrains product appearance consistency. Likewise, the long-term stability of the favorable coloration of pasta and biscuits remains unknown, and the industrial transformation of pitaya into baked products requires the resolution of critical bottlenecks, including pigment stabilization, process standardization, and cost controllability.

### 4.3 Natural pigment substitutes

Red-fleshed pitaya is a significant source of natural pigments and functional components (127, 128). Research on extraction technologies has focused on the recovery of bioactive substances from dried peels (129). Yellow-skinned pitaya peels, as by-products, are rich in betaxanthins (natural yellow-orange antioxidant pigments), and betaxanthin-rich extracts can be prepared using techniques such as spray drying (130). Red-skinned pitaya peels are abundant in betacyanins, which confer their characteristic red-purple coloration and are regarded as potential sources of natural pigments for the food and cosmetics industries (77). Pigment concentrates extracted from red-fleshed pitaya peels (e.g., microfiltered red-purple pitaya concentrate) exhibit vivid coloration and biological activity (127). Microfiltered red-purple pitaya pigment concentrate maintains stable microbiological, physicochemical, and chemical properties under refrigerated storage conditions (131). Furthermore, betacyanin extracts enriched from red-fleshed pitaya peels and flowers preserved deep pink coloration in tagliatelle and meringue cookies without significantly altering the product texture during a 14-day storage period (124). However, the 14-day storage period represents only short-term laboratory validation and does not encompass long-term shelf-life studies under ambient temperature, light exposure, or actual retail conditions. Microencapsulation and other technological approaches have been employed to enhance stability (132). For instance, betacyanins encapsulated with maltodextrin and gum arabic demonstrated improved stability during processing while retaining antioxidant capacity (133, 134). Additionally, studies have successfully developed gelatin-based soft candies with antioxidant activity using pure pitaya pulp (135). Although this approach can partially ameliorate betacyanin degradation, encapsulation efficiency, release kinetics, and sensory impacts remain unoptimized, and production costs increase.

### 4.4 Development of biocomposite materials

The substantial quantities of by-products, such as peels, generated during pitaya processing are rich in polysaccharides, phenolic substances, and natural pigments, and are regarded as potential resources for the development of functional materials (136). Studies have confirmed that pitaya peels can serve as sustainable raw materials for preparing eco-friendly active packaging materials and edible films (93). White-fleshed pitaya peel pectin was employed as a film-forming matrix, combined with betacyanins extracted from the peel as active components, to successfully develop a novel active edible film with antioxidant and indicator functions. The incorporation of montmorillonite as a reinforcing filler further enhanced the mechanical properties and barrier characteristics of the film (137). The recovery and reuse of compounds from by-products (such as peels) in the food and pharmaceutical industries align with the principles of the circular economy (138, 139). Additionally, mucilage extracted from yellow-skinned pitaya peels serves as a useful structural material in spray-drying processes (130).

## 5 Discussion and future perspectives

### 5.1 Variations in bioactive components across pitaya tissues, mechanisms of action, and industrial applications

The chemical composition of pitaya exhibits marked tissue specificity and cultivar-dependent variation across the entire plant. Red-fleshed cultivars are abundant in betacyanins, which exert antioxidant effects by scavenging reactive oxygen species and activating specific signaling pathways (70). White-fleshed cultivars are characterized by polysaccharide accumulation, and the specific glycan side-chain structures in the RG-I region of their pectin are closely associated with prebiotic activity (140). Functional complementarity across different tissues is also notable. The peel contains the highest levels of polyphenols, flavonoids, and dietary fiber (141). The flesh, rich in polyphenols, polysaccharides, and betacyanins, confers distinct advantages for natural pigment development and antioxidant applications (12). Although seeds constitute a minimal proportion of the fruit, they exhibit significantly higher levels of unsaturated fatty acids and polyphenols than the flesh (142). Triterpenoids and flavonol glycosides in the stems and flowers endow non-edible tissues with immunomodulatory and anti-inflammatory activities (15).

Current research has preliminarily revealed the multi-target and multi-pathway regulatory characteristics of pitaya (93). However, existing mechanistic studies have predominantly yielded correlational results. Alterations in the expression of key pathways, such as Nrf2 and NLRP3, have been inconsistent across different models. Moreover, the dose-transformation relationship between the *in vitro* free radical scavenging capacity and *in vivo* biological effects remains poorly understood.

The application of pitaya in the food industry demonstrates a trend toward diversification, although technological maturity varies considerably. Fermented products can enhance polyphenol bioaccessibility and generate characteristic flavors; however, fermentation parameters lack standardization, and interactions between acidic components and milk proteins may compromise product stability (143, 144). In natural pigment applications, the thermal instability of betacyanins leads to color loss during processing, whereas microencapsulation entails high costs and suboptimal encapsulation efficiency (133, 145, 146). Active packaging materials possess both antioxidant and indicator functions; however, breakthroughs in their mechanical properties and cost-effectiveness of scaled production remain necessary (147, 148).

### 5.2 Research limitations and future directions

#### 5.2.1 Clinical evidence

Current bioactivity evidence is derived almost exclusively from *in vitro* experiments and animal models, with a notable absence of rigorously designed randomized controlled human clinical trials. However, the effective dosage, bioavailability, long-term safety, and population-specific variations remain undefined. Future research should prioritize human intervention studies and establish efficacy evaluation frameworks based on biomarkers, including fasting blood glucose, inflammatory factor profiles, and gut microbiota metagenomics.

#### 5.2.2 Component stability

The thermal, light, and pH sensitivity of betacyanins restricts their application in thermally processed foods. Future efforts should focus on the development of novel encapsulation materials, modulation of release kinetics, impact of non-thermal processing technologies on active component retention rates, and synergistic stabilization mechanisms between betacyanins and other natural pigments.

#### 5.2.3 By-product utilization

Peels and seeds, as processing by-products, have yet to achieve a balance between extraction yield, purity, and cost. Current laboratory extraction methods are energy-intensive and unsuitable for large-scale industrial production. Future research should focus on developing low-energy continuous extraction-purification technologies and establishing quality grading standards for by-product raw materials to achieve precise matching across “raw material–process–product” chains (149, 150).

#### 5.2.4 Mechanistic elucidation

Existing research remains largely confined to microbial community composition analysis, with insufficient investigation of functional genes, metabolic pathways, and host–microbe interactions (e.g., gut–liver axis, gut–lung axis) (151). Future studies should integrate multi-omics technologies to identify key functional strains and explore personalized nutritional intervention strategies (152).

## 6 Conclusion

Pitaya possesses both nutritional and functional properties, and the efficient utilization of its whole-plant resources aligns with the prevailing demand in the food industry for natural and sustainable raw materials. However, the translation of fundamental research to industrial applications still requires overcoming multiple obstacles, including the absence of clinical validation, non-standardized process parameters, insufficient stability of active components, and cost control constraints. Future research should focus on an in-depth investigation of the mechanisms of action of active components, optimization of novel extraction and delivery technologies, and development of functional foods based on standardized evaluation systems. These efforts will fully unlock the comprehensive value of whole-plant pitaya resources in the fields of nutrition, health, and food industry. They will promote the transformation of pitaya from a fresh fruit into functional food ingredients, natural additives, and bioactive materials. This transition will provide a paradigm for the high-value utilization of tropical agricultural resources.
